# Single-shot structural analysis by high-energy X-ray diffraction using an ultrashort all-optical source

**DOI:** 10.1038/s41598-017-16477-0

**Published:** 2017-11-30

**Authors:** R. Rakowski, G. Golovin, J. O’Neal, J. Zhang, P. Zhang, B. Zhao, M. D. Wilson, M. C. Veale, P. Seller, S. Chen, S. Banerjee, D. Umstadter, M. Fuchs

**Affiliations:** 1 Department of Physics and Astronomy, University of Nebraska–Lincoln, Lincoln, Nebraska 68588 USA; 2 STFC Rutherford Appleton Laboratory, Harwell, Didcot, Oxfordshire OX11 0QX UK

## Abstract

High-energy X-rays (HEX-rays) with photon energies on order of 100 keV have attractive characteristics, such as comparably low absorption, high spatial resolution and the ability to access inner-shell states of heavy atoms. These properties are advantageous for many applications ranging from studies of bulk materials to the investigation of materials in extreme conditions. Ultrafast X-ray diffraction allows the direct imaging of atomic dynamics simultaneously on its natural time and length scale. However, using HEX-rays for ultrafast studies has been limited due to the lack of sources that can generate pulses of sufficiently short (femtosecond) duration in this wavelength range. Here we show single-crystal diffraction using ultrashort ~90 keV HEX-ray pulses generated by an all-optical source based on inverse Compton scattering. We also demonstrate a method for measuring the crystal lattice spacing in a single shot that contains only ~10^5^ photons in a spectral bandwidth of ~50% full width at half maximum (FWHM). Our approach allows us to obtain structural information from the full X-ray spectrum. As target we use a cylindrically bent Ge crystal in Laue transmission geometry. This experiment constitutes a first step towards measurements of ultrafast atomic dynamics using femtosecond HEX-ray pulses.

## Introduction

Experiments using HEX-rays with photon energies on order of 100 keV can access information that is complementary to that obtained by lower-energy hard X-ray methods, which typically operate at photon energies around 10 keV. In particular, due to the comparatively low photo-absorption cross-section, HEX-rays have a high penetration depth which allows the investigation of bulk materials^1^ or solids and liquids in extreme conditions^2^, such as under high pressures and at high densities^3^ or at high temperatures^4^. The large momentum of HEX photons can be used in scattering to access information that cannot be obtained with lower-energetic X-rays. More specifically, the large momentum transfer in HEX diffraction experiments enables measurements with a significantly higher spatial resolution and the almost flat Ewald sphere allows the observation of a large part of reciprocal space spanning several Brillouin zones in a single geometry. This can be of particular advantage in measurements of phonon distributions via diffuse scattering experiments^1^.

Tunable HEX-ray beams are routinely generated using synchrotron emission from relativistic electron beams that propagate through dipole magnets or permanent-magnet wigglers^5^. The wavelength of the emitted radiation can be adjusted over a wide range by varying the energy of the electron beam^6^. However, the pulse duration of storage-ring based synchrotron facilities is typically limited to 30–100 ps, which is not sufficiently short to perform studies of ultrafast dynamics on the atomic temporal scale. HEX-ray pulses with a duration of 20 ps have been generated using a more compact source that is based on inverse Compton scattering of optical photons (laser wiggler) from a relativistic electron beam^7^. X-ray pulses with sub-picosecond duration using storage-ring based sources have been generated by sophisticated electron beam manipulations^8^ and by a perpendicular Compton scattering geometry^9^ however at the cost of greatly reduced photon flux. Recently, X-ray free-electron lasers (XFELs)^10,11^ that are capable of ultrashort X-ray pulses with enormous intensities have become available. However, current facilities cannot reach high-energy X-ray photon energies.

Here, we use an all-optical inverse Compton scattering source where both, the relativistic electron beam and the optical wiggler are generated by a laser^12^. The relativistic electron pulse is produced by a laser-wakefield accelerator, in which a high-power femtosecond laser pulse is focused into a gas target where it excites a plasma wave^13^. Electrons are accelerated by this wave to relativistic energies within a distance of only a few millimeters. This method can be used to drive an X-ray source with a relatively compact footprint. More importantly, it enables the possibility of investigating atomic dynamics on an ultrafast time scale as the generated electron bunches (and thus the subsequently generated X-ray pulses) are intrinsically only a few femtoseconds long with a perfect temporal synchronization to the driver laser^14,15^. However, these sources typically generate pulses with a comparatively broad bandwidth (a few tens of percent) and operate at a relatively low repetition rate on order of 1 Hz. An additional challenge resulting from the compact geometry of these sources is the background radiation that is mainly generated by scattering of the relativistic electron beam at the chamber walls and the beam dump as well as the highly noisy environment typically present at high-power laser interactions.

Here we demonstrate a technique that enables diffraction experiments using such beam parameters. To this end, we use HEX-rays to observe diffraction from the (220) reflection of a cylindrically bent Ge crystal. The crystal was used in a Laue transmission X-ray spectrometer geometry^16,17^. As detector we used a pixelated energy-resolving X-ray camera in single-photon counting mode^18^. In this arrangement, we were able to filter the diffraction signal in photon energy by using the energy resolution of the camera. The additional independent spectral information obtained by the spatial dispersion of the crystal diffraction allowed us to determine the crystal lattice constant despite significant background contamination. A similar energy-resolved approach based on powder diffraction has been proposed as in-line X-ray diagnostics^19^ and has been used as *in situ* shock compression diagnostics using a flat crystal^20^. We were able to demonstrate that through energy filtering of the diffraction signal, it is possible to use the full X-ray spectrum of ~50% (FWHM) for the determination of the crystal lattice spacing. Specifically, we were able to resolve the Ge(220) lattice spacing to be 2 Å with a spatial resolution of 2.1 pm (one standard deviation). We extended the method to demonstrate that we can measure the lattice constant using only a single pulse that contained just ~10^5^ X-ray photons in a 50% bandwidth at a central photon energy of 90 keV. Single-shot determination of the lattice constant is of particular interest for experiments that measure systems with many degrees of freedom, such as investigations of time-resolved dynamics that require measurements at many different time steps or for experiments that investigate non-reversible excitations. Since in our case the lattice constant is well known, we also used the method to retrieve the source spectrum with minimal background contamination.

## Results

### Experimental Setup

**Figure 1:**
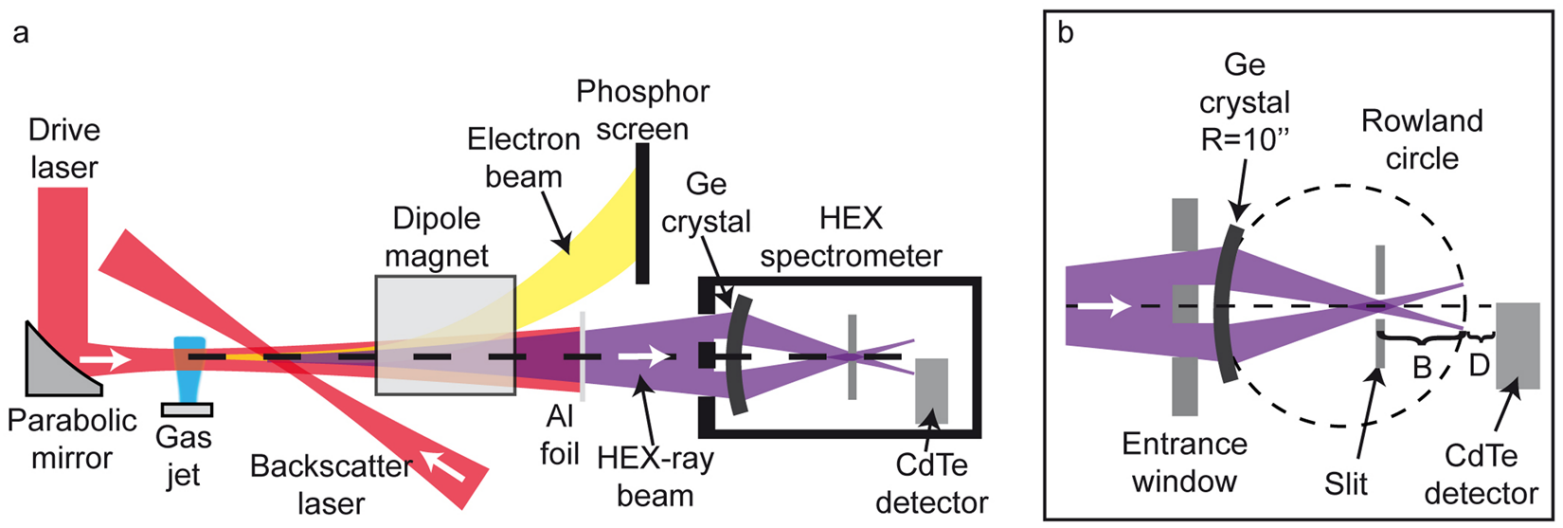
Schematic of the experimental setup. (**a**) shows the schematic of the inverse Compton X-ray source. The X-rays are generated by backscattering of a high-intensity laser beam from a relativistic electron beam that is produced by a laser-plasma interaction. Subsequently, the electrons are bent out of the X-ray path by a dipole magnet and the optical laser pulse is filtered out by a thin Al foil, while the X-rays are transmitted. The spectrum of the electron beam is diagnosed with a phosphor screen. A cylindrically bent Ge crystal is positioned at 2.25 m downstream of the X-ray source. The diffraction signal is observed with an energy-resolving pixelated CdTe X-ray camera. (**b**) shows an enlarged schematic of the HEX-ray spectrometer showing the bent Ge crystal with radius of curvature (ROC) of 0.254 m and the detector. The (220) planes of the crystal are used for diffraction. The crystal is enclosed in a housing with a lead entrance window that blocks the direct beam except for a small pinhole. A slit is placed at the position where the diffracted X-rays cross the spectrometer axis in order to block any direct line-of-sight between the source and the detector. The X-rays are detected in a plane close to the Rowland circle indicated by the dashed line. The distance of the slit to the focal circle is indicated by *B* and the distance of the detector position from the focal circle by *D*. The whole spectrometer is shielded from background through a lead and Teflon enclosure.

For the experiment, we used an ultrafast all-optical X-ray source^12^. The X-rays were produced by inverse Compton scattering of an optical laser beam (800 nm) from a relativistic electron beam, which was generated using laser-wakefield acceleration (for more details see methods). The source is capable of generating up to 10^6^ photons per shot and for this experiment was operated at a central photon energy of ~90 keV with a relative energy spread of ~50% (FWHM). The X-ray beam has a divergence on order of ~10 mrad and a shot-to-shot pointing fluctuation of ~10 mrad. As target we used a 0.16 mm thick cylindrically bent Ge crystal with a radius of curvature (ROC) of 0.254 m located in air at a distance of 2.25 m from the X-ray source (see Fig. 1a). The Ge crystal is placed in a Cauchois-type transmission spectrometer setup^21^. In this geometry, X-rays of equal energy that are diffracted by the crystal from a position close to the spectrometer axis are focused onto a circle (Rowland circle) with a diameter that is equal to the ROC of the bent crystal^16^. The spectral dispersion *X*_*D*_ of photon energy *E* on a flat detector located a distance *D* behind the focal circle is given by^21^

1 $${X}_{D}\approx nhc\frac{R}{2{d}_{latt}E}(1+\frac{D}{B}),\,$$

where *n* is the diffraction order, *h* the Planck constant, *c* the speed of light, *d*_*latt*_ the crystal lattice spacing of the used reflection, *R* the crystal radius of curvature and *B* the distance from the slit to focal circle (see Fig. 1b). The dispersion distance *X*_*D*_ from the spectrometer axis scales linearly inverse with the photon energy *E*. We used the (Laue) reflection of the (220) planes of a Ge crystal, for which *d*_*latt*_ ~2 Å. As detector we used a pixelated energy-resolving high energy X-ray HEXITEC camera based on a CdTe crystal^18^. It has an active area of 20 × 20 mm^2^ with 80 × 80 pixels each with a size of 250 × 250 µm^2^. In order to work in single-photon counting mode, the detector was located at 2.5 m from the source.

### Photon-energy resolved dispersion measurement

**Figure 2:**
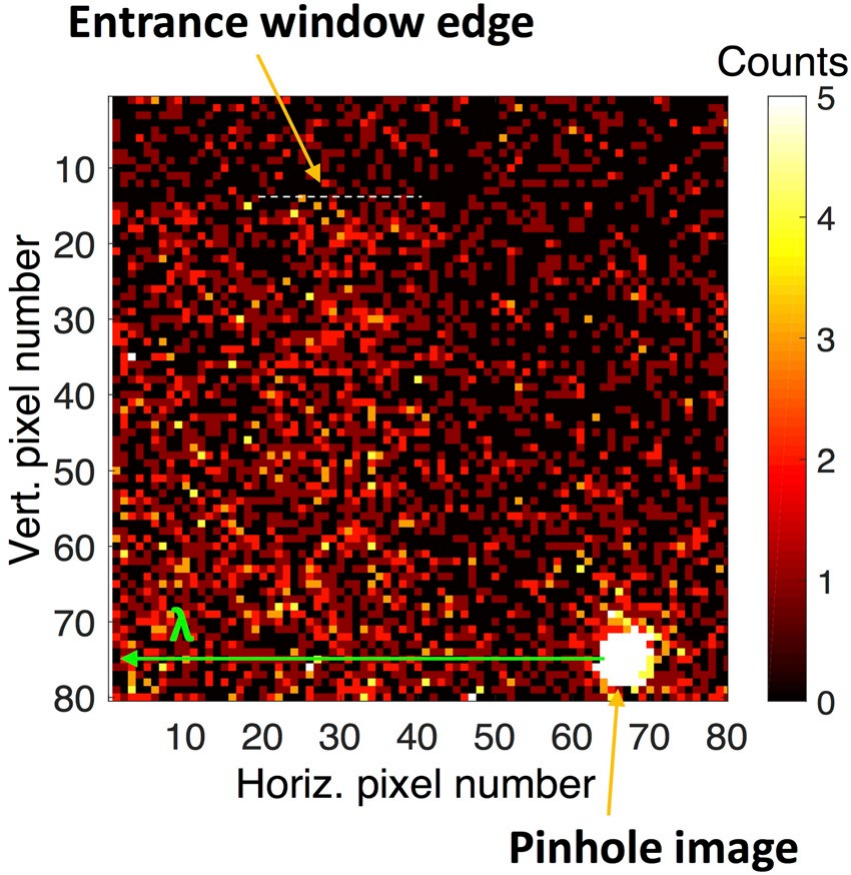
Raw X-ray diffraction signal. The figure shows the X-ray diffraction signal from the (220) Laue reflection of a bent Ge crystal, summed over consecutive 87 shots. The crystal spectral dispersion direction is horizontal. The top edge of the detector chip was blocked by the spectrometer entrance window, which is shown by the white dotted line. The signal above this line is indicative of the background. On the lower right a small part of the undispersed direct beam that is transmitted through a pinhole in the entrance window can be seen.

The diffraction signal was recorded for every X-ray shot. The total signal obtained by the summation of 87 consecutive shots is shown in Fig. 2. The lower-right corner of the image shows the undispersed direct beam transmitted through a pinhole in the spectrometer entrance window. Due to the finite chip size only a fraction of the dispersed beam was recorded.

**Figure 3:**
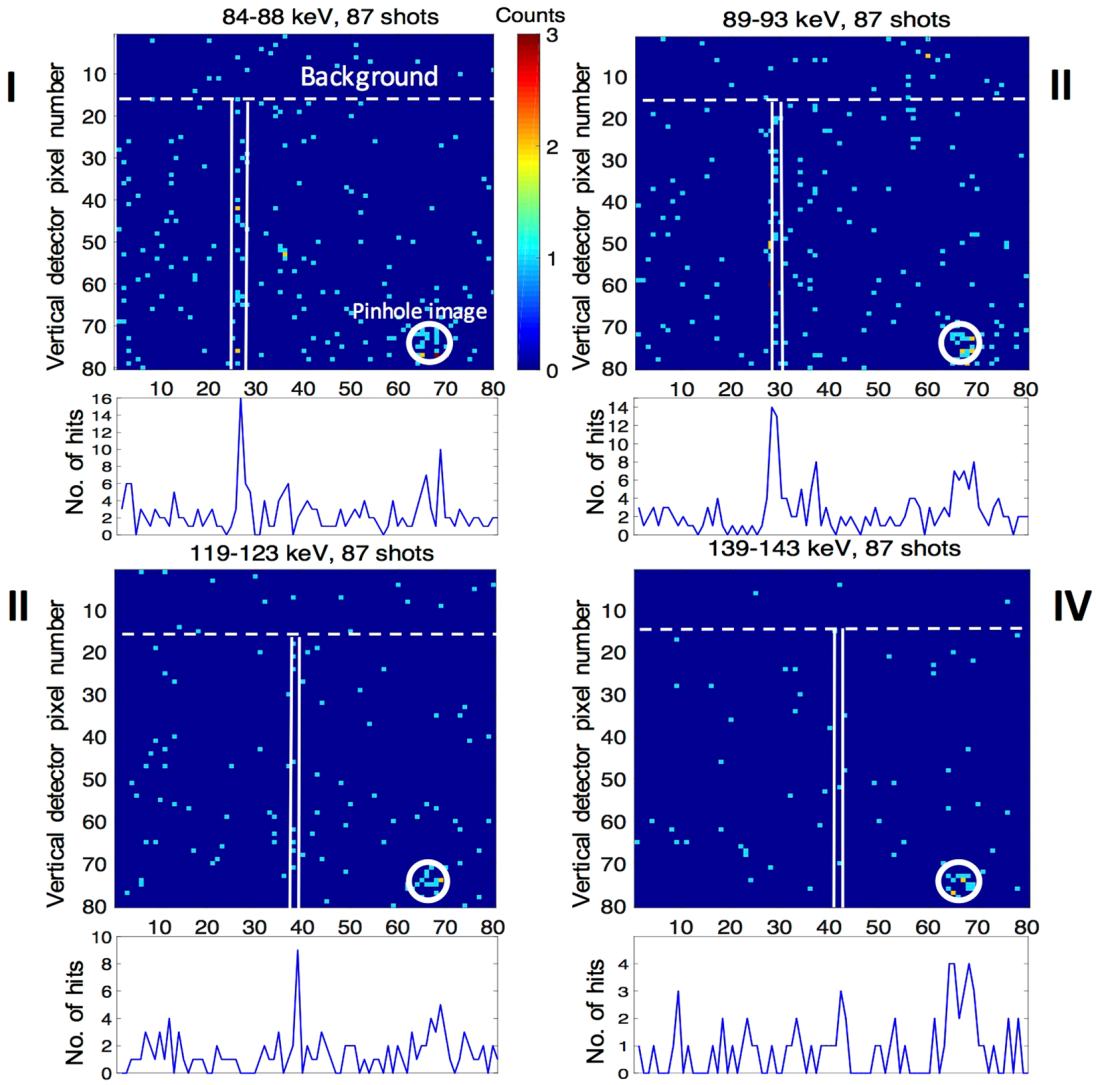
Hitmap of photon-energy filtered diffraction signal. The observed diffraction signal is separated into narrow spectral bands using the photon-energy selectivity of the camera. The figure shows spatial photon hit maps for bands at 84–88 keV (I), 89–93 keV (II), 119–123 keV (III), and 139–143 keV (IV). The vertically integrated signal can be seen below each image. The solid white lines indicate the theoretical position according to the spectral dispersion of the crystal diffraction given by equation (1). The signal above the white dotted line, where the X-ray beam was blocked by the entrance window, gives an estimate on the background. Each individual shot is processed and then summed. The figure shows the combined signal of 87 shots. The undispersed direct beam transmitted through a pinhole in the entrance window can be seen on the lower right corner of each image.

**Figure 4:**
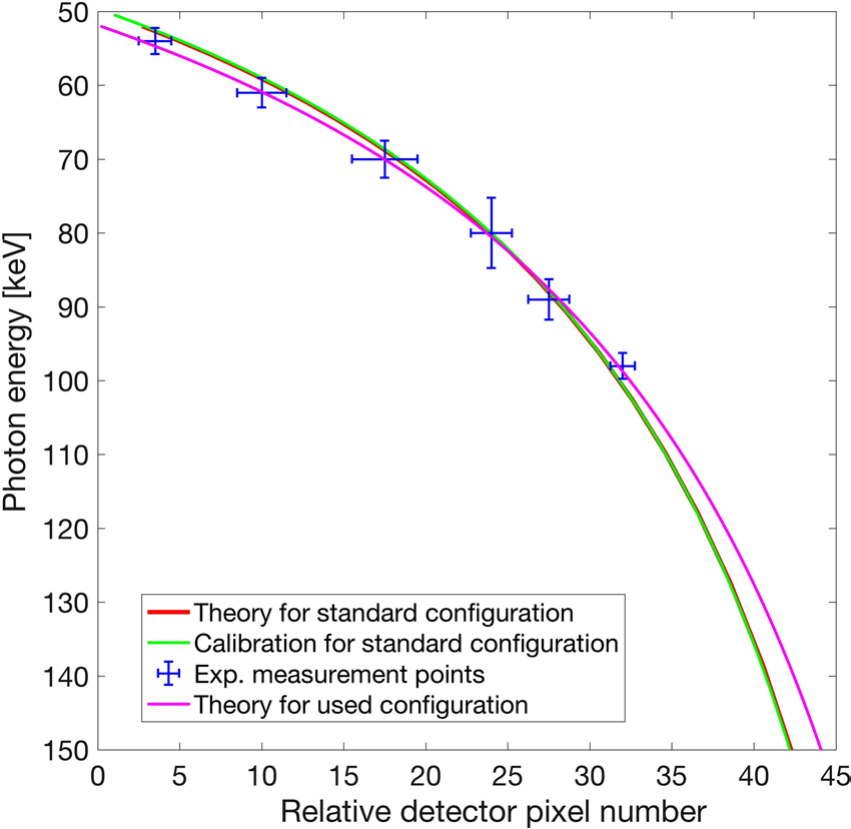
Spectrometer dispersion curve. The figure shows a comparison between the experimentally observed positions of the energy-filtered peaks and the crystal spectral dispersion. The blue data points indicate the peak positions of the vertically integrated energy-filtered camera images for selected energies. The horizontal error bars are the FWHM-widths of the integrated peak and the vertical error bars the widths of the selected energy band. The curves for the calibration of the bent-crystal spectrometer in a standard configuration with the detector located directly on the Rowland circle (*D* = 0) is shown in green and the theoretical curve according to equation (1) in red. Note that the two curves almost completely overlap. The magenta curve shows the spectrometer dispersion adjusted for the configuration used in this experiment, which includes a small offset of the X-ray camera position from the Rowland circle (*D*/*B* = 0.11) and a small rotation of the Ge crystal (see methods).

At the photon energy range used in the experiment, the X-ray camera has an intrinsic energy-resolution of ~1 keV (FWHM)^18^, which allows us to spectrally filter the observed signal. By using the combined information of the energy resolution of the X-ray detector and the spatial dispersion of the crystal diffraction we can significantly increase the signal to noise ratio. To this end, for each individual shot we spectrally filter the recorded images into narrow energy bands in a range of 50–150 keV using the camera energy resolution. We integrate the filtered images along the vertical (non-dispersion) dimension. An example of this method for selected energy bands can be seen in Fig. 3. To verify the validity of this method, we compare the peak of the integrated lineout that we have obtained for each energy band with the expected spectral dispersion given by equation (1). Figure 4 shows the excellent agreement of the camera energy-selected peaks with the theoretical dispersion curve adjusted for our spectrometer setup (see methods).

### Retrieved source spectrum

**Figure 5:**
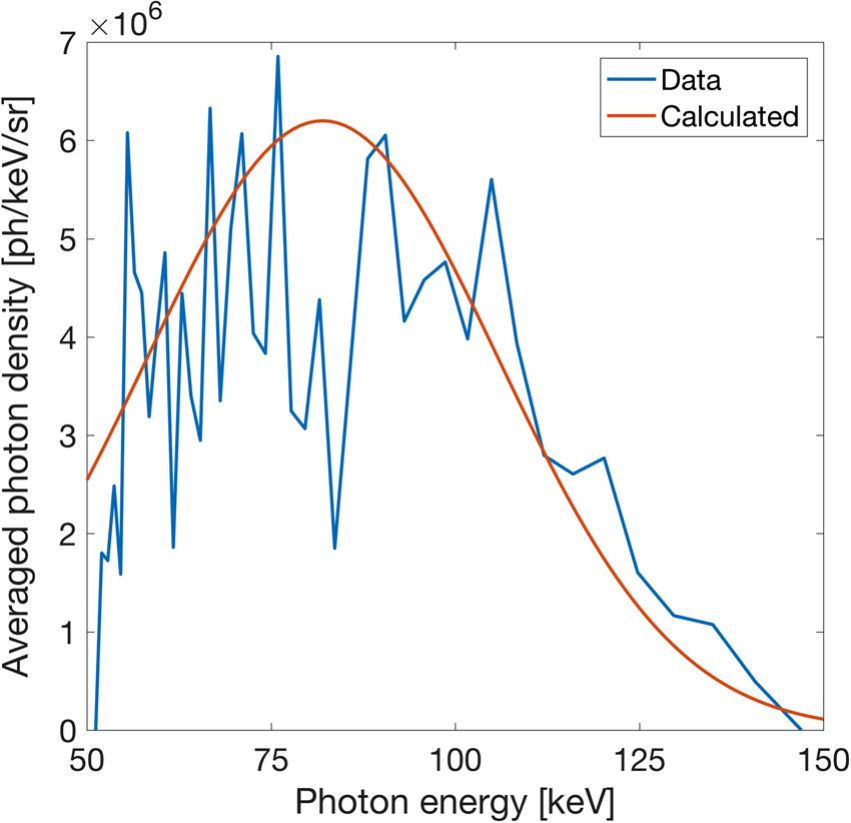
Measured double-differential spectrum of the inverse Compton X-ray source. The spectrum is generated by post-processing each shot individually and then averaging over 87 shots (blue). The expected spectral shape (red) is calculated for a 5% energy spread in the laser beam and an electron beam with 5% energy spread, 5% shot-to-shot energy fluctuation and a 10 mrad divergence. The retrieved spectrum takes into account the diffraction efficiency of the Ge(220) reflection, the detector efficiency and the transmission through the beamline (see methods). The detector observes an effective solid angle of ~5 × 10^–5^ sr. Due to the divergence and pointing fluctuations of the X-ray beam, on average only a fraction of the whole beam is detected.

Since in our case the lattice constant of the crystal is well known, we can use the energy-filtered diffraction signal to determine the spectrum of the X-ray source. In principle, the source spectrum can be obtained from either the crystal dispersion or by using the energy-resolution of the X-ray camera. However, the recorded diffraction images contain a significant background that is mainly due to the interaction of the relativistic electron beam with the beam dump and is visible despite massive shielding of the detector. The comparison of background to signal can be approximately estimated from the photon-energy filtered images shown in Fig. 3. The background can be measured by only operating the electron accelerator while blocking the backscattering laser pulse, such that no inverse Compton scattering X-rays are generated. However, it is challenging to correct for in a single shot as it strongly varies in both, energy and spatial distribution from shot to shot. In order to obtain a spectrum with significantly decreased background contamination, we use the combined information obtained from the spatial dispersion due to the crystal diffraction and the camera energy-filtered signal. We filter out the background in the data post-processing by correlating the spatial position of a photon on the detector with its energy. To this end, we select a narrow photon energy-band with a width according to the camera resolution (~1 keV), spatially filter the image according to the crystal spectral dispersion given by equation (1) and then vertically integrate (perpendicular to spectral dispersion direction) the signal. Extending this method over the whole energy range allows us to retrieve the spectral photon density of the source (see Fig. 5). In order to obtain the source photon number, we have taken into account the integrated Laue diffraction efficiency of the Ge(220) reflection extracted from Fig. 6a of ref.^21^ for each photon energy (see methods) and the beamline transmission. Note that on average we detect only part of the total X-ray beam. This is because the solid angle of the fraction of the crystal, for which the diffraction pattern is detected by the camera is ~5 × 10^−5^ sr and the beam divergence and shot-to-shot pointing fluctuation each are on order of 10 mrad in the horizontal and vertical direction. By using a long propagation distance of ~2.5 m between the source and the detector we sufficiently decrease the photon flux on the camera to operate in single-photon counting mode. Regardless, our method is robust to photon pile-up, a process where two photons that are simultaneously detected by the same pixel appear as a single photon of higher energy. In case of pile-up, the camera would detect a higher photon energy, which would skew the measured shape of the spectrum. However, due to the additional discrimination of the spatial location of the detected photons, in our case pile-up does not distort the measurement.

**Figure 6:**
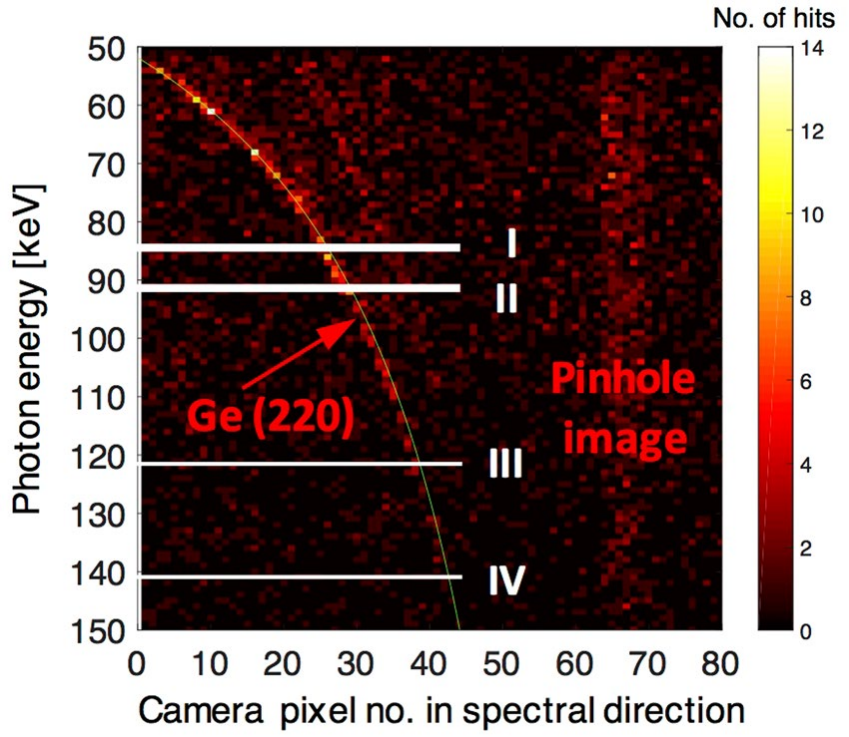
Photon-energy resolved diffraction signal. The figure shows the spatially dispersed diffraction pattern from the bent Ge crystal resolved by photon energy. Each row is obtained by filtering the signal into ~1 keV bands using the photon-energy resolution of the X-ray camera and then vertically integrating the image, similar as shown in Fig. 3. The energy ranges shown in Fig. 3 (I–IV) are indicated by white lines. A clear correlation signal can be observed for a lattice spacing that corresponds to the Ge(220) reflection, despite significant background. The theoretical curve for our experimental conditions according to equation (1) is shown in green (see methods). The figure shows the signal summed over 87 shots. The energy-resolved direct beam that is transmitted through a pinhole in the entrance window of the spectrometer can be seen as vertical stripe centered around the 67^th^ pixel.

### Determination of the Ge(220) lattice constant

We can also use this method to determine the lattice constant of the crystal. In particular, the correlation of the photon-energy filtered data with the crystal spectral dispersion allows us to get a high-resolution measurement of the lattice parameter, even from a single shot with only ~10^5^ photons and a comparably broad spectrum of ~50% (FWHM). Figure 6 shows the photon-energy resolved diffraction pattern for the sum of 87 shots. The figure has been generated by energy-filtering the total signal using the X-ray camera resolution and vertically integrating the image for each energy. Note that unlike for the extraction of the source spectrum, in this case the signal is not filtered by the horizontal dispersion position. Despite the noisy background, a clear diffraction signal from the Ge(220) planes can be seen. This method allows us to perform structural analysis of the crystal lattice using the full bandwidth of the X-ray spectrum.

**Figure 7:**
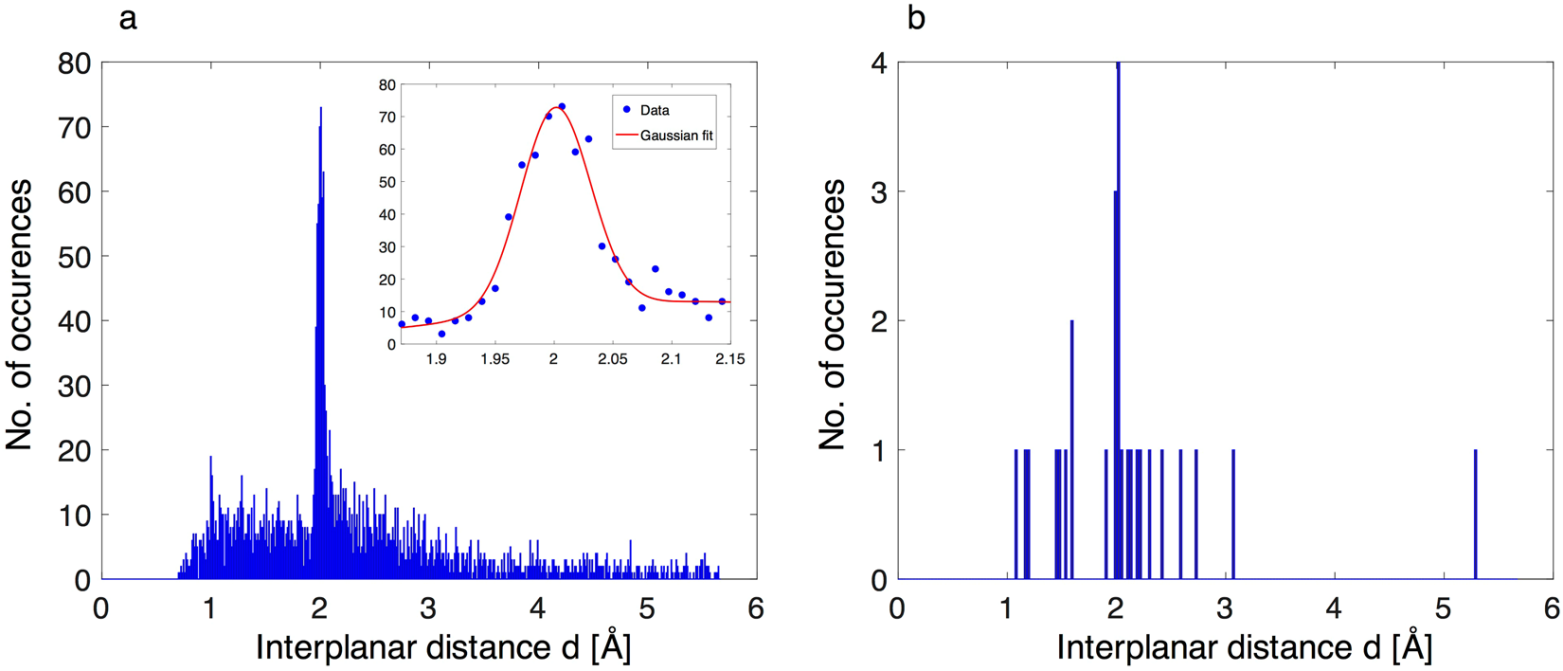
Histogram of measured effective lattice constant. (**a**) Histogram of 87 X-ray shots. The crystal lattice constant is determined from the photon-energy resolved diffraction pattern. For each detected photon, the photon energy *E* and the spatial position *X*_*d*_ are deduced. An effective lattice constant *d* is determined using equation (1). The peak at *d* = 2 Å corresponds to the lattice spacing of the Ge(220) planes. By fitting a Gaussian distribution (inset), we find a standard deviation of 2.1 pm. The histogram is generated from the sum of 87 shots, the bin width is 1.1 pm (total 500 bins). (**b**) Histogram of a single shot with bin width of 2.8 pm.

In order to infer the lattice constant of the crystal from the photon-energy resolved diffraction signal we determine for each detected photon its energy *E* and spatial position *X*_*d*_. Through equation (1), we can determine an effective lattice constant *d* for each photon. We perform this analysis for every shot. The histogram for all 87 shots can be seen in Fig. 7a. The known lattice parameter for Ge is *d* = 5.658 Å and for the (220) planes is expected to be  ${d}_{220}=d/\sqrt{{h}^{2}+{k}^{2}+{l}^{2}}\approx 2.0{\AA }$, where *h*, *k* and *l* are the Miller indices of the reflection. The histogram shows a peak well above background at 2.0 Å. From the histogram, we can infer a measurement precision of 2.1 pm (1 standard deviation) for all 87 shots, corresponding to the resolution of our measurement. The main sources of measurement uncertainties are the accuracies of the determination of the spatial positions of the detected photons due to the finite width of the camera pixels of 250 µm and the finite camera energy resolution of ≲1 keV for our photon energy range. The inter-planar distance is determined to be *d* = 2 ± 0.021 Å.

### Single-shot Determination of Ge lattice constant

We also demonstrate that this method allows us to determine the lattice constant with a high-resolution using a single shot with only ~10^5^ photons and a comparably broad spectrum of ~50% (see Fig. 7b). A clear peak well above background can be seen at the Ge(220) lattice spacing. The bin width for the histogram has been chosen to be on order of the measurement uncertainty that has been determined from the sum of 87 shots.

## Discussion

We have observed a diffraction signal from a bent single crystal using an ultrafast all-laser driven high-energy X-ray source operated at ~90 keV. By energy-filtering the diffraction signal using an energy-resolving HEX-ray camera, we were able to extract a nearly background free spectrum of the source. More importantly, we were able to demonstrate a technique that can take advantage of the full X-ray bandwidth for atomic-scale structural analysis. This allowed us to deduce the crystal lattice constant using only a single X-ray shot with just ~10^5^ photons and 50% bandwidth. This is of particular interest for experiments that require measurements at many different parameter points, such as investigations of the ultrafast time-evolution of structural dynamics. Despite significant background contamination, the measured signals were well above background. This approach can be beneficial for experiments that are performed in a highly noisy environment as often present in high-power laser interactions. The observed signal can be further enhanced by source developments and by decreasing the X-ray propagation distance to the sample. This is possible as the method is robust to photon pile-up. The method demonstrated here enables the use of novel ultrafast HEX-ray sources that typically generate a comparably low number of photons per shot in a broad bandwidth and operate at relatively low repetition rates for diffraction experiments. In a next step, we will extend this technique to flat crystal samples. In the future, we expect that our approach will enable time-resolved investigations of ultrafast dynamics using novel next-generation femtosecond high-energy X-ray sources.

## Methods

### All-optical Inverse Compton Scattering Source

The experiments were performed in the Extreme Light Laboratory at the University of Nebraska – Lincoln using the Diocles laser facility. The high-energy X-ray radiation is generated using an all-optical X-ray source. The X-rays were created through inverse Compton scattering of an optical (800 nm) laser beam from a relativistic electron beam, which was simultaneously generated using the same laser system. To this end, the laser beam is split into a driver for the electron accelerator and into a backscattering pulse. The driver pulse (1.4 J, 34 fs) is focused to an intensity of 5.4 × 10^18^ W/cm^2^ using an *f*/14 off-axis parabolic mirror and interacts with a gas jet to generate an electron beam via laser-wakefield acceleration. A dual gas-jet target has been used as accelerator for this experiment^22^. The X-rays are subsequently generated by the interaction of a backscattering laser pulse (0.3 J, 40 fs) focused to an intensity of 1 × 10^18^ W/cm^2^ with the electron beam in a nearly counter-propagating geometry. The source is capable of producing X-ray beams (~10^6^ photons per shot) with photon energies tunable in the range of a few tens of keV to around 10 MeV. Taking into account a 5% energy spread in the laser beam and an electron beam with 5% energy spread, 5% shot-to-shot energy fluctuation and a 10 mrad divergence results in a FWHM bandwidth of the X-ray pulse of $({\rm{\Delta }}\omega /\omega )\approx 67{\rm{ \% }}$. The photon energy can be increased by increasing the electron beam energy. For this experiment, the source was operated at a central photon energy of ~90 keV. Because of the comparably small normalized vector potential of the backscattering laser beam of *a*_0_ = 0.7, the radiation is mainly emitted into the fundamental peak centered at around 85 keV with a negligible harmonic content. The divergence of the X-ray beam is ~10 mrad and the shot-to-shot pointing fluctuations are also ~10 mrad.

### Cauchois-Type Transmission X-ray Spectrometer

A schematic of the spectrometer can be seen in Fig. 1b. The spectrometer uses a 0.16 mm thick cylindrically bent Ge crystal with a radius of curvature (ROC) of 0.254 m in Laue transmission geometry^21^. The X-ray detector was located at a distance of 2.5 m from the source. The spectrometer was aligned using the drive laser beam. X-rays of equal energy that are emitted from a point source and are incident onto the curved crystal at small angles are focused through Laue diffraction. The X-rays are focused onto a point on the focal circle (Rowland circle) with a diameter that is equal to the ROC of the crystal. The (220) reflection was used in this experiment. According to Bragg’s law, the diffraction angle depends on the photon energy and the spectral dispersion at a detector placed near the Rowland circle is linear with the photon wavelength. The detector is shielded by a lead entrance window and a slit close to the center of the focal circle, such that there is no direct line-of-sight between the source and the detector except for a 1 mm pinhole that indicates the spectrometer axis. The entrance window has two symmetric rectangular openings (each 30.5 mm high and 16.5 mm wide) separated by 6.5 mm opaque lead bar with the pinhole drilled in its center. In order to block any undiffracted radiation, a lead slit is placed where the diffracted polychromatic X-ray beam intersects the spectrometer axis (at a distance *B* = 12.7 cm from the back of the focal circle). The diffraction pattern was measured with a pixelated 2D energy-resolving X-ray camera. Due to experimental constraints, the camera was located at a distance of *D* = 1.4 cm from the Rowland circle. The spectrometer was previously calibrated using an image plate placed at *D* = 0 and a tungsten K-alpha X-ray source and K-edges of Tb (52 keV), Pb (88 keV) and U (115.6 keV) thin foils. For the analysis, a correction for the detector position of *D/B* = 1.11 has been used in equation (1). Furthermore, a small rotation of the Ge crystal has been taken into account as a constant offset of −1 × 10^6^ Å. In order to shield the detector from background radiation that is mainly generated by scattering of the relativistic electron beam at the chamber walls and the beam dump, the setup was enclosed a lead and Teflon housing.

### Energy-resolving Pixelated X-ray Detector

The HEXITEC X-ray camera developed in the UK by the Science and Technology Facilities Council (STFC, UK) uses a 1 mm thick CdTe detector. The chip is composed of an array of 80 × 80 square pixels (250 μm side) with a total area of 20.35 mm × 20.45 mm ref.^18^. The camera was recording continually using a 100 μs exposure. Only frames with a detectable pinhole image signal (plus one frame earlier and one later) were analyzed. The energy of each detected photon can be deduced from the deposited charge into the camera. The detector is sensitive to X-ray photon energies in the range of 4–200 keV with quantum efficiency of ~100%. In single-photon counting mode, the camera can spatially and spectrally resolve X-ray photons with an energy resolution of 0.8 keV at 59.5 keV (1.5 keV at 141 keV). Charge-sharing between pixels has been found to be negligible as an analysis between single pixels and binned 3 × 3 super-pixels has not shown any significant difference.

### Extracted Source Spectrum

The double-differential source spectrum ${d}^{2}I/(dEd\Omega )$ per solid angle *dΩ* and unit photon energy ${dE}$ can be determined from the measured signal at the detector *S(E, dE)* at photon energy *E*, the integrated reflectivity of the crystal *δ*(*E*), the quantum efficiency of the detector *D(E)* and the observed angle by the camera in the non-dispersive direction *θ*_*y*_ by

$$\frac{{d}^{2}I}{dEd\Omega }=\frac{S(E,dE)}{\delta (E)D(E){\theta }_{y}}.$$

In our case, the integrated efficiency of the Ge(220) reflection is *δ*(*E*) ~ 7 × 10^−6^ rad at 50 keV (3 × 10^−6^ rad at 100 keV)^21^, *D(E)* = 100% and *θ*_*y*_ = 6.4 mrad. The beamline transmission including a 8 mm borosilicate window has been taken into account.

### Data Availability Statement

The datasets generated during and analyzed during the current study are available from the corresponding author on reasonable request.
